# Impact of the introduction of an endotracheal tube attachment device on the incidence and severity of oral pressure injuries in the intensive care unit: a retrospective observational study

**DOI:** 10.1186/s12912-018-0274-2

**Published:** 2018-02-08

**Authors:** Jaye Hampson, Cameron Green, Joanne Stewart, Lauren Armitstead, Gemma Degan, Andrea Aubrey, Eldho Paul, Ravindranath Tiruvoipati

**Affiliations:** 1 Department of Intensive Care Medicine, Peninsula Health, Frankston Hospital, 2 Hastings road, Frankston, VIC 3199 Australia; 2 Department of Epidemiology and Preventive Medicine, School of Public health and Preventive Medicine, Faculty of Medicine, Nursing and Health Sciences, Monash University, Clayton, VIC Australia; 3 Clinical Haematology Department, The Alfred Hospital, Melbourne, Victoria 3181 Australia

**Keywords:** Pressure injury, Pressure wound, AnchorFast, Endotracheal intubation, Critical care

## Abstract

**Background:**

Endotracheal tube (ETT) fasteners such as the AnchorFast™ claim to assist with the prevention of oral pressure injuries in intubated patients, however evidence to support their clinical efficacy is limited. This retrospective observational study aimed to investigate the impact of the introduction of the AnchorFast™ device on the incidence of oral pressure injuries in mechanically ventilated patients.

**Methods:**

Data was collected from patient case notes and clinical incident reports for October 2010 to June 2013 (*pre-AnchorFast)* and July 2013 to March 2016 (*post-AnchorFast)*. Incidence and location of oral pressure injuries associated with securing device, and compliance with institutional policies related to reducing oral pressure injuries were recorded.

**Results:**

Incidence of oral pressure injuries increased from 1.53/100 intubated patients in the pre-AnchorFast period to 3.73/100 intubated patients in the post-AnchorFast period (IRR = 2.43, 95%CI = 1.35–4.38; *p* = 0.003). Across both study periods, patients with an ETT secured using AnchorFast™ had significantly increased risk of oral pressure injuries (IRR = 2.03, 95%CI = 1.17–3.51; *p* = 0.02). There was also a significant difference in location of pressure injuries sustained with ETTs secured using cloth tapes (53.6% in corner of the mouth) vs. AnchorFast™ (75% on the lips) (*p* = 0.008). Among patients with oral pressure injuries, compliance with institutional policies relating to the prevention of pressure injuries was significantly greater after the introduction of the AnchorFast™ (9.1% vs 64.5%, *p* = 0.004).

**Conclusions:**

The incidence of oral pressure injuries increased significantly following the introduction of the AnchorFast™ device. Further research is required to establish the reasons for this observed increase to and identify ways to reduce the risk of pressure injuries with ETT securement devices.

## Background

Pressure injuries are a leading cause of preventable harm to hospitalised patients worldwide, affecting between 1 and 11% of patients in acute-care settings [1]. Among all hospitalised patients the prevalence rate of pressure injury is highest for patients in Intensive Care Units (ICU) [2]. Globally, the incidence of pressure injuries among patients admitted to ICU ranges from 5 to 20%, with a prevalence of between 14 and 47% [3–5]. Patients in ICU have many unique factors that make them vulnerable to the development of pressure injuries. Risk factors for pressure injury development in critically ill patients may include impaired sensation, altered level of consciousness, reduced mobility, sedation, decreased tissue perfusion, nutritional compromise, and vasoactive medications [3, 6–8].

Medical devices are often an essential component in delivering necessary care to critically ill patients, yet are also increasingly being recognised as a potential cause of pressure injury. Medical device-related pressure injuries (MDRIs) are defined as a localised injury to the skin or underlying tissue as a result of sustained pressure from a device [9]. The majority of MDRIs occur on the head, neck and face [10], and may be caused by poor device fit, improper securement, or poor visualisation of the skin under the device making it difficult to perform skin assessment [9, 11]. A lack of practice guidelines and staffing workload and experience may also impact on the risk of a medical device related pressure injury [12]. The proportion of pressure injuries that are device-related varies in the literature from 10% to 40% [2, 6, 13]. Black et al. [9] reported that patients with a medical device were 2.4 times more likely to develop a pressure injury.

Although there is an increasing awareness of the risk of MDRIs, there are few studies that have addressed specific devices and their impact on the development of pressure injury. Two recent studies have found endotracheal tubes (ETT) and nasogastric tubes to be leading causes of MDRIs [13, 14], with intubated patients at risk of developing pressure injuries from the ETT and/or the methods or devices used to secure it [15]. ETTs are secured to prevent tube migration, and to avoid unplanned extubation. There are several methods for securing ETTs, including adhesive or cloth tapes, and endotracheal tube attachment devices (ETADs). These devices are designed specifically to hold the ETT securely in a way that facilitates regular repositioning of the ETT to prevent the development of pressure injuries caused by the tube resting on the inside of the mouth or lips for prolonged periods. These devices are therefore marketed as having the potential to reduce rates of oral pressure injuries [16]; however evidence to support these claims is limited [15, 17–20]. Two studies have found reduced skin breakdown with commercial ETT holders when compared to adhesive tape [19, 20]. Furthermore ETT holders were shown to significantly reduce internal and external movement of endotracheal tubes [20]. A systematic review and meta-analysis found significant reduction in lip excoriation with commercial devices (*p* < 0.001; OR = 0.2, 95%CI = 0.1–0.5), but no significant reduction in facial trauma (*p* = 0.11; OR = 0.4, 95%CI = 0.1–1.2) [17]. The degree of ETT displacement was found to be less with commercial devices than with adhesive tape in the setting of significant heterogeneity of the studies included [17]. A recent study comparing sixteen ETT securement methods using anatomically correct intubation models with embedded pressure sensors found that commercial devices exerted more pressure on the face than non-commercial devices and commercial ETT holders allowed for rapid and secure movement of ETT from one side of the mouth to the other. [15].

A commercially available ETAD, the AnchorFast™ (Hollister), was introduced into clinical practice in our department in mid-2013.

This study aimed to retrospectively evaluate the impact of the introduction of the AnchorFast™ device on the incidence of oral pressure injuries in our ICU.

## Methods

### Study setting

The study hospital ICU is a 15-bed university-affiliated non-tertiary Metropolitan medical and surgical ICU located in Victoria, Australia. Approximately 1100 patients are treated in this ICU each year, with about 40% requiring invasive mechanical ventilation (IMV).

AnchorFast™ devices were introduced into clinical practice in this unit from the start of July 2013. Prior to, and following the introduction of the AnchorFast™ into this department, nurses have received ongoing education in the proper use and placement of this device. Prior to the introduction of AnchorFast™, ETTs were secured using cloth tapes; after its introduction, both methods of ETT securement (AnchorFast and cloth tapes) were available for use. The choice of ETT securement method used in the post-AnchorFast period was at the discretion of the bedside nurse, with the exception of specific contraindications listed below.

Institutional pressure injury prevention guidelines state that cloth tapes should be changed and ETT repositioned every 6 h, or when the cloth is soiled; while the ETTs should be repositioned every 2 h for patients with an AnchorFast™ in situ. AnchorFast™ devices should be replaced every 3–5 days as per manufacturers’ instructions or clinical need. Repositioning of the ETT and replacement of tapes or AnchorFast™ devices should also be clearly documented on the patient’s ICU flow chart. Pressure injuries are reported using the Victorian Health Incident Management System, a database for the collection of clinical incidents and adverse events. Severity of pressure injuries is graded by the nurse generating the report, according to the current National Pressure Ulcer Advisory Panel (NPUAP) staging system [21].

### Study design

This was a retrospective observational study, investigating the incidence of reported pressure injuries to the mouth and lips of patients, prior-to and following the introduction of the AnchorFast™ device into clinical practice The time period from 01/10/2010–31/06/2013 was defined as the ‘pre-AnchorFast’ period; while 01/07/2013–31/03/2016 was defined as the ‘post-AnchorFast’ period.

### AnchorFast™ Endotracheal tube attachment device

**Fig. 1:**
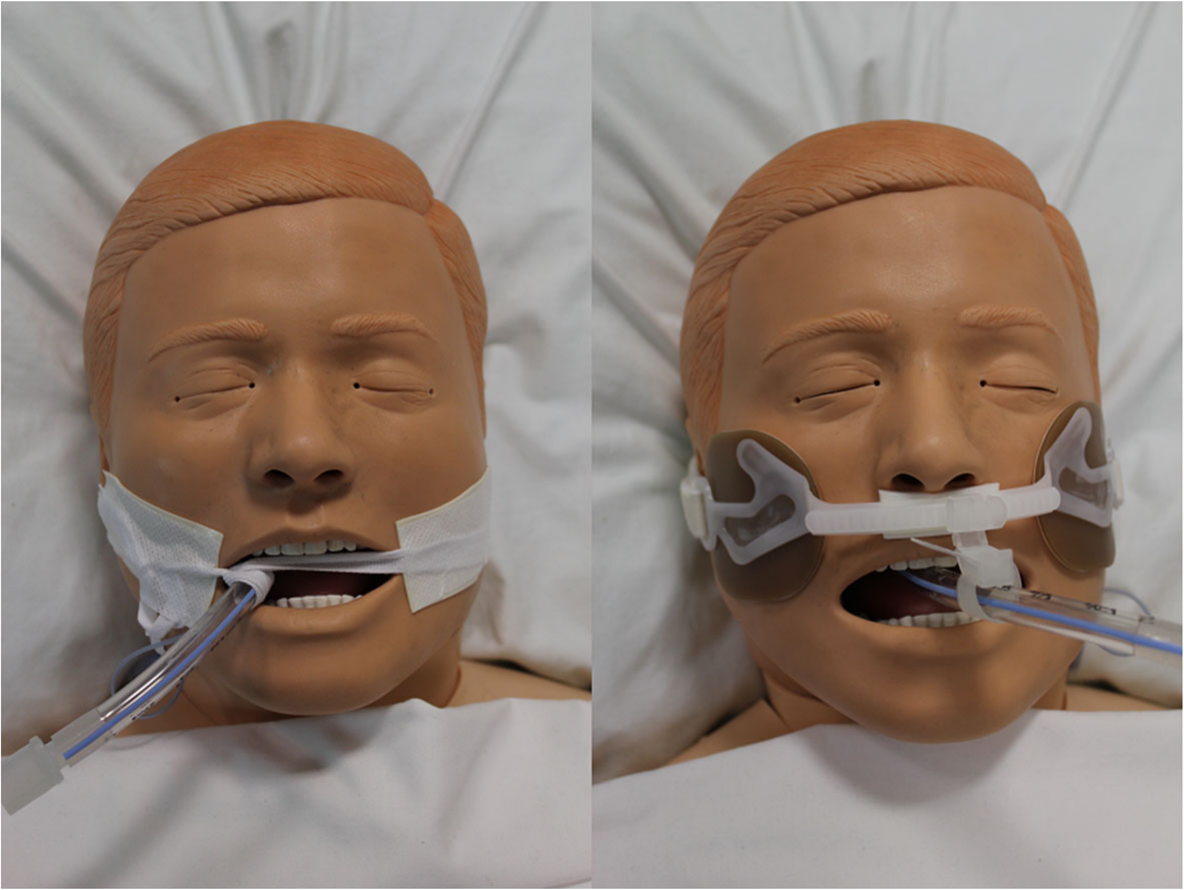
Two methods of endotracheal tube (ETT) securement used in the study hospital. Cloth tapes (left) are looped around the ETT and tied around the patient’s head, passing under their ears. A foam dressing is placed between the cloth tape and the patient’s skin, and an adhesive tape is used to secure it in place. The AnchorFast™ device is shown on the right

The AnchorFast™ device is produced by Hollister (Hollister Incorporated, Libertyville, IL USA). AnchorFast™ (Fig. 1*)* includes a number of features aimed at reducing the occurrence of pressure injuries to the mouth and lips, such as a ‘gliding tube shuttle’ which enables the ETT to be easily repositioned while being held securely in place, and a ‘lip stabiliser’ that prevents the ETT from resting on the patient’s upper lip.

AnchorFast™ devices are contraindicated for patients without teeth, with facial oedema, or with protruding teeth, facial hair, profuse diaphoresis, or allergic reaction to the device’s skin barrier pads.

### Data collection

The total number of admissions to the study ICU, number of patients receiving mechanical ventilation, and the total hours of mechanical ventilation required by patients during each study period was retrieved from ICU patient databases. The medical records of all patients receiving invasive mechanical ventilation in the study ICU during the post-AnchorFast period were reviewed to identify whether patients ever had their ETT secured using an AnchorFast™ device.

All pressure injuries reported during the study period were retrieved from Victorian health incident management system (VHIMS). These reports were reviewed by one of the investigator (JH), and pressure injuries to the mouth or lips were identified. The date and time of pressure injury documentation, and severity and location of the pressure injury was retrieved from VHIMS reports. Location of pressure injuries was classified as ‘corner of mouth’, ‘lip’ (for injuries on the outer surface of the lip), or ‘mouth’ (for injuries on the inner surface of the mouth).

The case notes for patients with reported oral pressure injuries were reviewed. The following variables were collected: length of ICU and hospital stay; length of mechanical ventilation; location of intubation (e.g. ambulance, emergency department, ICU); method of ETT securement at intubation; ICU admission diagnosis; comorbidities; use of long term steroids, Adult Physiology and Chronic Health Evaluation (APACHE-III) illness severity score; Waterlow pressure injury risk score at ICU admission and prior to pressure injury documentation; and whether there was documentation of compliance with institutional pressure injury prevention guidelines in the 48 h prior to pressure injury documentation.

### Ethical considerations

This study was reviewed and approved as an audit by Research Governance of Peninsula Health (ref.: QA/16/PH/8). As this was a retrospective study requiring no patient contact, no informed consent was required.

### Statistical analysis

Continuous variables were assessed for normality and expressed as mean (standard deviation) or median (inter-quartile range) depending on the distribution of data. Categorical variables were summarised using frequencies, presenting the subject counts and percentages. Comparisons between groups were made using Wilcoxon rank-sum test for continuous variables and chi-square or Fisher’s exact test as appropriate for categorical variables. The incidence of pressure injuries were compared using Poisson regression with results reported as incidence rate ratios (IRR) and 95% confidence intervals. All calculated *p* values were two-tailed and *p* < 0.05 indicated statistical significance. Analyses were performed with Stata software version 14 (StataCorp, College Station, Texas, USA).

## Results

### Incidence of oral pressure injuries

Throughout the study period 2008 patients received invasive mechanical ventilation in ICU (1043 pre-AnchorFast, 965 post-AnchorFast). There were a total 230 pressure injury incident reports, of which 22.6% (*n* = 52) were oral pressure injuries to the mouth or lips (16 pre-AnchorFast, 36 post-AnchorFast). During the post-AnchorFast period, 61.8% (*n* = 596) of mechanically ventilated patients had their ETT secured with an AnchorFast™.

Throughout both study periods, there were 42 patients with 52 reported oral pressure injuries.

There was no significant difference between the pre- and post-AnchorFast periods in patient age, gender distribution, ICU or hospital length of stay, Waterlow score on ICU admission, risk factors for pressure injury development, and length of mechanical ventilation (Table 1). There was a significant difference in compliance with institutional policies aimed at preventing pressure injuries between the two periods. There was greater documented compliance with pressure injury prevention protocols in the 24 h prior to pressure injury documentation for patients in the post-AnchorFast period compared to those in the pre-AnchorFast period (64.5% vs. 9.1%; *p* = 0.004).

**Table 1:** Comparison of characteristics of patients with pressure injuries between pre- and post-AnchorFast periods

Variable	All patients (*N* = 42)	Pre-AnchorFast (*n* = 11)	Post-AnchorFast (*n* = 31)	*p*-value
Age (median, IQR)	56 (47.7–72.6)	52.2 (37.7–63.0)	56.7 (47.8–73.6)	0.43
Male Sex (%, n)	66.7% (28)	81.8% (9)	61.3% (19)	0.28
Malnourished at ICU admission (%, n)	19.0% (8)	36.4% (4)	12.9% (4)	0.17
Peripheral vascular disease (%, n)	2.4% (1)	9.1% (1)	0% (0)	0.26
Diabetes (%, n)	14.3% (6)	27.3% (3)	9.7% (3)	0.31
Current Smoker (%, n)	26.2% (11)	27.3% (3)	25.8% (8)	1.00
Long-term steroid therapy (%, n)	16.7% (7)	9.1% (1)	19.4% (6)	0.65
Serum albumin	34.5 (27.8–39.0)	30.0 (26.0–39.0)	35.0 (29.0–39.0)	0.45
Restricted mobility (%, n)	26.2% (11)	27.3% (3)	25.8% (8)	1.00
Waterlow score at ICU admission (median, IQR)	24 (17–28)	23.5 (14–24.5)	24 (17–28)	0.28
Length of mechanical ventilation, days (median, IQR)	7.6 (3.8–15.2)	8.7 (6.7–16.9)	6.9 (3.0–14.0)	0.10
ICU length of stay, days (median, IQR)	9.9 (4.6–21.2)	9.4 (7.8–23.1)	10.1 (4.2–20.9)	0.13
Hospital length of stay, days (median, IQR)	27.0 (11.6–36.3)	31.6 (14.9–38.3)	20.9 (10.6–36.3)	0.35
APACHE-III Score (median, IQR)	92.5 (72.8–111.3)	86.0 (73.0–121.0)	96.0 (69.0–107.0)	0.71
Time from intubation to PI reporting, days (median, IQR)	3 (1.9–6)	5 (2–6)	3 (1–7)	0.50
ICU Mortality (%, n)	14.3% (6)	18.2% (2)	12.9% (4)	0.64
Hospital Mortality (%, n)	28.6% (12)	27.3% (3)	29.0% (9)	1.00
Pressure injury severity				
1	21.4% (9)	18.2% (2)	22.6% (7)	1.00
2	78.6% (33)	81.8% (9)	77.4% (24)	

Similarly, there was no difference in patient demographics between patients that developed pressure injuries with AnchorFast devices or cloth tape in situ across study periods (Table 2). Those with AnchorFast™ devices in–situ at the time of the pressure injury report were significantly more likely to have documented compliance with pressure injury prevention protocols within the previous 24 h of pressure injury development, compared to those with cloth tapes in-situ at the time of pressure injury documentation (81.0% vs 19.0%; *p* < 0.001).

**Table 2:** Comparison of characteristics of patients with pressure injuries based on ETT securement method

Variable	All patients (*N* = 42)	Tape (*n* = 21)	AnchorFast (*n* = 21)	*p*-value
Age (median, IQR)	56 (47.7–72.6)	53.2 (39.2–67.1)	58.6 (49.3–73.6)	0.22
Male Sex (%, n)	66.7% (28)	81.0% (17)	52.4% (11)	0.10
Malnourished at ICU admission (%, n)	19.0% (8)	28.6% (6)	9.5% (2)	0.24
Peripheral vascular disease (%, n)	2.4% (1)	4.8% (1)	0% (0)	1.00
Diabetes (%, n)	14.3% (6)	23.8% (5)	4.8% (1)	0.18
Current Smoker (%, n)	26.2% (11)	19.0% (4)	33.3% (7)	0.48
Long-term steroid therapy (%, n)	16.7% (7)	9.5% (2)	23.8% (5)	0.41
Serum albumin	34.5 (27.8–39.0)	36.0 (27.0–39.5)	32.0 (27.5–36.0)	0.26
Restricted mobility (%, n)	26.2% (11)	28.6% (6)	23.8% (5)	1.00
Waterlow score at ICU admission (median, IQR)	24 (17–28)	23.5 (15.3–26.0)	25.0 (17.5–28.0)	0.20
Length of mechanical ventilation, days (median, IQR)	7.6 (3.8–15.2)	7.1 (3.8–16.7)	7.9 (3.6–13.9)	0.10
ICU length of stay, days (median, IQR)	9.9 (4.6–21.2)	8.31 (4.1–21.8)	10.7 (4.9–20.8)	0.51
Hospital length of stay, days (median, IQR)	27.0 (11.6–36.3)	26.0 (8.6–33.7)	28.0 (13.8–60.8)	0.36
APACHE-III Score (median, IQR)	92.5 (72.8–111.3)	93.0 (72.5–119.5)	92.0 (71.5–101.5)	0.41
Time from intubation to PI reporting, days (median, IQR)	3 (1.9–6)	3 (1–5.5)	4 (2–9)	0.12
ICU Mortality (%, n)	14.3% (6)	19.0% (4)	9.5% (2)	0.66
Hospital Mortality (%, n)	28.6% (12)	19.0% (6)	28.6% (6)	1.00
Pressure injury severity				
1	21.4% (9)	23.8% (5)	19.0% (4)	1.00
2	78.6% (33)	76.2% (16)	81.0% (17)	

The incidence of reported oral pressure injuries was 1.53 per 100 mechanically ventilated patients during the pre-AnchorFast period, compared to 3.73 oral pressure injuries per 100 mechanically ventilated patients in the post-AnchorFast period (IRR: 2.43, 95% CI: 1.35–4.38; *p* = 0.003). Across both study periods, the incidence of oral pressure injuries among those with ETTs secured with tape was 1.98 per 100 ventilated patients, compared to 4.03 per 100 ventilated patients with an AnchorFast™ (IRR: 2.03, 95% CI: 1.17–3.51; *p* = 0.02). The incidence of oral pressure injuries by time period and method of ETT securement is shown in Table 3*.*

**Table 3:** Incidence of oral pressure injuries by study period (pre- vs. post- introduction of the AnchorFast™ device), and by method of ETT securement (cloth tapes vs. AnchorFast™ device) across both study periods

	All patients	Pre-AnchorFast period	Post-AnchorFast Period	Tape ETT securement	AnchorFast ETT securement
Number of Mechanically ventilated patients	2008	1043	965	1412	596
Total Ventilation Hours	201,152	93,602	107,550	109,711	91,441
Number of Oral Pressure Injuries reported	52	16	36	28	24
Oral Pressure Injuries per 100 ventilated patients	2.59	1.53	3.73*	1.98	4.03^#^
Oral pressure Injuries per 10,000 ventilation hours	2.59	1.71	3.35^	2.55	2.63^ǂ^

* p = 0.003; # p = 0.02; ^ *p* = 0.03; ^ǂ^ *p* = 0.92

Across both study periods, there was a statistically significant difference in the location of oral pressure injuries between those sustained with an AnchorFast™ device or cloth tape in-situ (*p* = 0.008). Those with an ETT secured using AnchorFast™ devices were most likely to sustain injuries to their lip (75%); while patients with their ETT secured using cloth tape were most likely to sustain injuries to the corner of their mouths (53.6%) (Table 4*)*.

**Table 4:** Location of oral pressure injuries across study period, by method of ETT securement (cloth tapes vs. AnchorFast™ device) at the time of pressure injury report

	ETT secured using cloth tape	ETT secured with AnchorFast device	*p*-value
Number of pressure Injuries reported	28	24	
Location of Pressure Injury (%, n)			
Corner of mouth	53.6% (15)	20.8% (5)	**0.008**
Lip	32.1% (9)	75% (18)	
Inside of lip or mouth	14.3% (4)	4.2% (1)	

There was no significant difference in the time from intubation to pressure injury development between patients with tape and AnchorFast™ ETT securement (median 3 vs 4 days respectively; *p* = 0.12). There was also no significant difference in the severity of pressure injuries sustained with tapes (median = 2, range = 1–2) vs AnchorFast™ devices (median = 2, range = 1–2; *p* = 0.71). A majority of injuries in both groups were assessed as being stage 2 (tape = 76.2%, AnchorFast = 81.0%).

## Discussion

Medical devices are a leading cause of pressure injuries in critically ill patients [2, 6], with ETTs and nasogastric tubes accounting for the majority of these [13, 14]. Oral pressure injuries related to endotracheal intubation accounted for 22.6% of all documented pressure injuries over a 6.5-year period in our ICU.

The incidence of reported oral pressure injuries appears to have increased significantly following the introduction of the AnchorFast™ device in our unit. Overall, patients with an AnchorFast™ in place were approximately twice as likely to develop oral pressure injuries. To our knowledge, only one previous study has investigated the impact of the introduction of the AnchorFast™ device on the incidence of oral pressure injuries. This retrospective 20-month study found a decrease in incidence of oral pressure injuries following the introduction of the AnchorFast™ device, from an incidence of 1.25 injuries per 100 ventilated patients to 0.06/100 ventilated patients [22]. These findings are at odds with those of the present study. It is unclear where this discrepancy may arise from, however variations in staffing and patient mix may contribute. In particular, Zaratkiewicz and colleagues reported an average of over 300 patients per month receiving IMV [22], compared to an average of 26 patients per month in our unit during the study period. This is likely to influence staffing experience and workload, as well as the length of mechanical ventilation that patients received. No information regarding patient acuity, length of mechanical ventilation, or staffing was provided in this article to enable comparisons with the present study.

Compliance with institutional policies aimed at reducing oral pressure injuries improved significantly following the introduction of the AnchorFast™ device. Similarly, compliance was significantly better among patients that developed oral pressure injuries with their ETT secured using an AnchorFast™ device compared to patients with cloth tape during the post-AnchorFast period.

According to promotional materials, the design of the AnchorFast™ device allows nursing staff to more easily reposition the ETT. This is likely to have contributed to the improved compliance with pressure injury prevention policies observed in this study; however, this does not appear to relate to a decrease in pressure injury incidence when implemented in routine practice. The reasons for this warrant further investigation.

The location of pressure injuries to the mouth and lips varied significantly between those sustained with AnchorFast™ devices and cloth tapes in place. The locations of pressure injuries reported with each method reflect areas of pressure exerted by these devices on the underlying skin (see Fig. 1), and these findings therefore have implications for the prevention of pressure injuries using these devices. This may include adjusting the way that cloth tapes are secured to reduce pressure across the corners of the mouth, and potential modifications to the AnchorFast™ device.

The severity of oral pressure injuries did not differ between those sustained with tapes and AnchorFast™ devices, with the majority of injuries reported as being stage 2 or “partial thickness skin loss with exposed dermis” [21]. The appropriateness of using standard pressure wound staging systems for the assessment of oral pressure wounds has previously been questioned [23].

### Limitations

This study has a number of limitations that must be acknowledged. As it was conducted at a single centre the findings may have been influenced by a number of aspects of clinical practice that vary between centres, including staffing and patient characteristics.

The incidence of oral pressure injuries over the study period was relatively low, at 2.59 injuries per 100 invasively-ventilated patients. This limited the sample size available to compare the characteristics of patients who developed oral pressure injuries on the basis of their method of ETT securement.

As this is a retrospective observational study causality cannot be clearly established for the increased incidence in oral pressure injuries observed following the introduction of the AnchorFast™ device. It is possible that this finding also reflects an increased awareness and reporting of pressure injuries following the introduction of this device; however within the post-AnchorFast period, where both securement methods were used, two-thirds of oral pressure injuries occurred with AnchorFast™ devices in situ. In addition, after adjusting for the proportion of all mechanically ventilated patients who received each method of ETT securement, those with AnchorFast™ devices were found to be at significantly increased risk of oral pressure injuries. This is despite no significant differences in risk factors for pressure injuries between those that developed oral pressure injuries with cloth tapes or AnchorFast™ devices.

The retrospective nature of this study limits discussion to those pressure injuries that were documented. Clinical practice guidelines state that all pressure injuries must be reported appropriately, and all nursing staff in this unit received regular training in the identification and reporting of pressure injuries. Despite this, it is possible that some pressure injuries were never documented. In addition, assessment of nursing compliance with pressure injury prevention guidelines relied upon existing documentation.

Finally, despite being highly statistically significant, effect size confidence intervals were relatively large for comparisons of oral pressure injuries between the pre- and post-AnchorFast periods (IRR = 2.43; 95% CI = 1.35–4.38), and between patients with AnchorFast devices and cloth tapes (IRR = 2.13; 95% CI = 1.17–3.51).

Despite these limitations, this study represents a significant contribution to the existing body of literature regarding the utility of the AnchorFast™ device (and ETADs more broadly) to prevent oral pressure injuries associated with endotracheal intubation.

### Recommendations

These findings highlight the importance of continually evaluating the efficacy of medical devices, particularly where there is limited empirical evidence to support their use. Given the present findings, the lack of available evidence, and the limitations of retrospective observational studies, a prospective randomised controlled trial may be warranted to investigate whether ETADs demonstrate any benefit for the prevention of oral pressure injuries when compared to cloth tape. A cost-benefit analysis may also be of relevance, given the increased financial costs associated with such devices.

## Conclusion

Incidence of oral pressure injuries increased significantly following the introduction of the AnchorFast™ ETT securement device. Patients with an ETT secured using an AnchorFast™ device were found to be at a significantly increased risk of developing oral pressure injuries.

## Abbreviations

95% CI: 95% Confidence Interval
APACHE-III: Adult Physiology and Chronic Health Evaluation illness severity score, version three
ETAD: Endotracheal tube attachment device
ETT: Endotracheal tube
ICU: Intensive Care Unit
IMV: Invasive mechanical ventilation
IRR: Incidence rate ratio
MDRI: Medical device-related injury
NPUAP: National Pressure Ulcer Advisory Panel
VHIMS: Victorian health incident management system
